# High pretransplant hepcidin levels are associated with poor overall survival and delayed platelet engraftment after allogeneic hematopoietic stem cell transplantation

**DOI:** 10.1002/cam4.974

**Published:** 2016-12-01

**Authors:** Soichiro Sakamoto, Hiroshi Kawabata, Junya Kanda, Tatsuki Uchiyama, Chisaki Mizumoto, Toshiyuki Kitano, Tadakazu Kondo, Masakatsu Hishizawa, Naohisa Tomosugi, Akifumi Takaori‐Kondo

**Affiliations:** ^1^ Department of Hematology and OncologyGraduate School of MedicineKyoto UniversityKyotoJapan; ^2^ Department of Hematology and ImmunologyKanazawa Medical UniversityUchinada‐machiJapan; ^3^ Department of Hematology and ImmunologyJapanese Red Cross Otsu HospitalOtsuJapan; ^4^ Division of Advanced MedicineMedical Research InstituteKanazawa Medical UniversityUchinada‐machiJapan

**Keywords:** Allogeneic hematopoietic stem cell transplantation, engraftment, hepcidin, iron

## Abstract

Iron overload is considered a risk factor for mortality in patients with hematopoietic malignancies. Hepcidin is a key regulator of systemic iron balance. We previously reported dynamic changes of serum hepcidin‐25 levels in patients with hematologic malignancies after allogeneic hematopoietic stem cell transplantation (allo‐HSCT). In this study, we retrospectively analyzed the association of pretransplant hepcidin‐25 levels with overall survival (OS), engraftment, and other clinical outcomes of allo‐HSCT in patients with hematologic malignancies. A total of 166 patients were divided into two groups depending on their pretransplant serum hepcidin‐25 levels; their median age was 49.5 years, and the median follow‐up time was 46.8 months. At 3 years, the patients in the high‐hepcidin group had a significantly lower OS than those in the low‐hepcidin group (49.2 vs. 69.0%, respectively; *P* = 0.006). Multivariate analysis revealed that pretransplant serum hepcidin‐25 level, sex, and disease status were independently associated with OS. The incidence of platelet engraftment was significantly lower in the high‐hepcidin group than in the low‐hepcidin group, whereas no significant differences were observed in neutrophil and reticulocyte engraftments between these groups. Hence, pretransplant serum hepcidin levels can be a marker for predicting delayed platelet recovery after allo‐HSCT.

## Introduction

Iron is a double‐edged sword; its deficiency causes anemia due to underproduction of hemoglobin, whereas its excess can cause organ damage through overproduction of reactive oxygen species 1. Therefore, the levels of iron in the body are maintained within a narrow range by sophisticated mechanisms in healthy individuals. The key regulator of iron balance in the body is hepcidin, a small peptide mainly produced by hepatocytes. Hepcidin was originally discovered as an antimicrobial peptide in urine 2; eventually, its presence in human serum was demonstrated using mass spectrometry‐based techniques 3. Hepcidin secreted from hepatocytes binds directly to ferroportin, the only known iron exporter in the mammalian cells, inducing the latter's internalization and degradation and thereby reducing iron efflux from macrophages, hepatocytes and enterocytes 4. Among various forms of mature hepcidin peptides detected in serum, hepcidin‐25 is the major form that is responsible for the degradation of ferroportin 2, 5.

We previously reported dynamic changes of serum hepcidin‐25 levels in patients with hematologic malignancies after allogeneic hematopoietic stem cell transplantation (allo‐HSCT) 6. Pretransplant hepcidin‐25 levels of these patients were slightly elevated; levels increased further during conditioning treatments and reached their peak on approximately day 14 before gradually decreasing 6. Our data indicated that serum hepcidin‐25 levels were inversely correlated with hematopoietic activity in the setting of allo‐HSCT. We also studied the relationship between pretransplant serum hepcidin‐25 levels and the outcomes of allo‐HSCT by analyzing 55 patients with hematologic malignancies, and found that elevation of the former was associated with an increased risk of early bacterial infections 7. However, there was no significant association between pretransplant serum hepcidin‐25 elevation and the overall survival (OS) of these patients within 100 days after allo‐HSCT, possibly due to the small number of participants and the short follow‐up period. In this study, we investigated the association of pretransplant serum hepcidin‐25 levels with OS, engraftment, and other clinical outcomes of allo‐HSCT in a greater number of participants, and with a longer follow‐up period.

## Patients and Methods

### Study population

We retrospectively reviewed the medical records of 177 adult patients who underwent their first allo‐HSCT for hematologic malignancies at Kyoto University Hospital between July 2006 and August 2013. A total of 166 patients whose pretransplant serum hepcidin‐25 level data were available, including 55 patients who were enrolled in our previous study 7, were included in the analyses. This study was performed in accordance with the Helsinki Declaration and approved by the Ethics Committee of Kyoto University Graduate School and Faculty of Medicine. Written informed consent was obtained from all the participants.

### Serum analysis

Serum samples were obtained in the morning prior to the administration of conditioning regimen and stored in tubes at −80°C until analysis. Serum hepcidin‐25 levels were measured using a liquid chromatography‐tandem mass spectrometry‐based assay system 3. Other serum parameters were measured using standard laboratory techniques.

### Statistical analysis

The primary endpoint was the impact of pretransplant serum hepcidin‐25 levels on overall survival after allo‐HSCT. The secondary endpoints were the impacts of the same on the incidences of relapse, nonrelapse mortality, grades G2–4 acute graft‐versus‐host disease (GVHD), as well as recovery of neutrophils (≥500/*μ*L), reticulocytes (≥1%), and platelets (≥50,000/*μ*L without transfusion support) after allo‐HSCT; the competing event in the cumulative incidence analyses was defined as death or second allo‐HSCT without recovery.

The patients were divided into two groups depending on their pretransplant levels of serum hepcidin‐25 or ferritin. The cutoff points for the hepcidin‐25 and ferritin levels were set at their median values. The patient and transplant characteristics between the two groups were compared using the Mann–Whitney test or Fisher's exact test, as appropriate. Standard‐risk disease was defined as complete remission in cases of acute myeloid leukemia, acute lymphoblastic leukemia, malignant lymphoma, and plasma cell myeloma; untreated or complete remission in cases of myelodysplastic syndrome and myeloproliferative disorder; and chronic phase in cases of chronic myeloid leukemia. High‐risk disease was defined as any other disease state. The conditioning regimen was categorized as either myeloablative or reduced intensity according to the National Marrow Donor Program and the Center for International Blood and Marrow Transplant Research operational definitions 8.

Overall survival was estimated using the Kaplan–Meier method, and groups were compared using the log‐rank test. Cumulative incidences of relapse, nonrelapse mortality, grade 2–4 acute GVHD, and engraftment of neutrophils, reticulocytes, and platelets were compared between groups using Gray's test 9.

The Cox proportional‐hazard model was applied to assess the factors that potentially influenced the study endpoints. The following items were considered confounders: serum hepcidin levels (<35 vs. ≥35 ng/mL), the age of the recipient (<50 vs. ≥50 years), the sex of the recipient, diagnosis (myeloid or lymphoid malignancies), risk of disease (standard or high risk), source of stem cells (HLA‐matched‐ or HLA‐mismatched‐related donor graft, unrelated bone marrow and peripheral blood, or unrelated cord blood), ABO matching (matched or mismatched), conditioning regimen (myeloablative or reduced intensity), and prophylaxis against GVHD (tacrolimus‐ or cyclosporine‐based). For multivariate analysis, stepwise backward selection was performed keeping hepcidin‐25 and variables with *P* values <0.1. *P* < 0.05 was considered statistically significant.

All the analyses were conducted using EZR (Saitama Medical Center, Jichi Medical University, Saitama, Japan), which is a graphical user interface for R (The R Foundation for Statistical Computing, version 2.13.0) 10.

## Results

### Characteristics of patients and transplants

The characteristics of the patients and transplants are shown in Table 1. The median pretransplant serum hepcidin‐25 level was 35 ng/mL, which was higher than that of healthy volunteers (7.8 ± 7.0 ng/mL, *n* = 63). Patients were divided into two groups (*n* = 83 each) according to the pretransplant serum hepcidin‐25 levels: the low‐hepcidin group (<35 ng/mL) and the high‐hepcidin group (≥35 ng/mL). There were no significant differences in patient and transplant characteristics between the low‐ and high‐hepcidin groups (Table 1).

**Table 1:** Characteristics of patients and transplants

Variables	Total*n* = 166	Hepcidin‐25, <35 ng/mL*n* = 83	Hepcidin‐25, ≥35 ng/mL*n* = 83	*P* value
Age at transplant (years)				
Median (range)	49.5 (17–66)	47 (17–65)	51 (19–66)	0.22
Sex				
Male	92	48	44	0.64
Female	74	35	39	
Disease				
Myeloid malignancies	103	49	54	0.52
Lymphoid malignancies	63	34	29	
Risk of disease				
Standard	100	53	47	0.43
High	66	30	36	
Source of stem cells				
HLA‐matched related	31	18	13	0.66
HLA‐mismatched related	13	5	8	
Unrelated BM or PB^a^	75	38	37	
Unrelated CB	47	22	25	
ABO blood‐type matching				
Matched	67	32	35	0.75
Mismatched	99	51	48	
Conditioning regimen				
Myeloablative intensity	79	45	34	0.12
Reduced intensity	87	38	49	
GVHD prophylaxis				
Tacrolimus‐based	143	68	75	0.18
Cyclosporine‐based	23	15	8	

a Among the stem cell sources from unrelated donors, all but 1 were BM.

Patient and transplant characteristics between two groups were compared using the Mann–Whitney *U*‐test or Fisher's exact test, as appropriate. HLA, human leukocyte antigen; BM, bone marrow; PB, peripheral blood; CB, cord blood; GVHD, graft‐versus‐host disease; tacrolimus‐based, tacrolimus with or without other agents; cyclosporine‐based, cyclosporine with or without other agents.

### OS, relapse, and nonrelapse mortality

**Figure 1:**
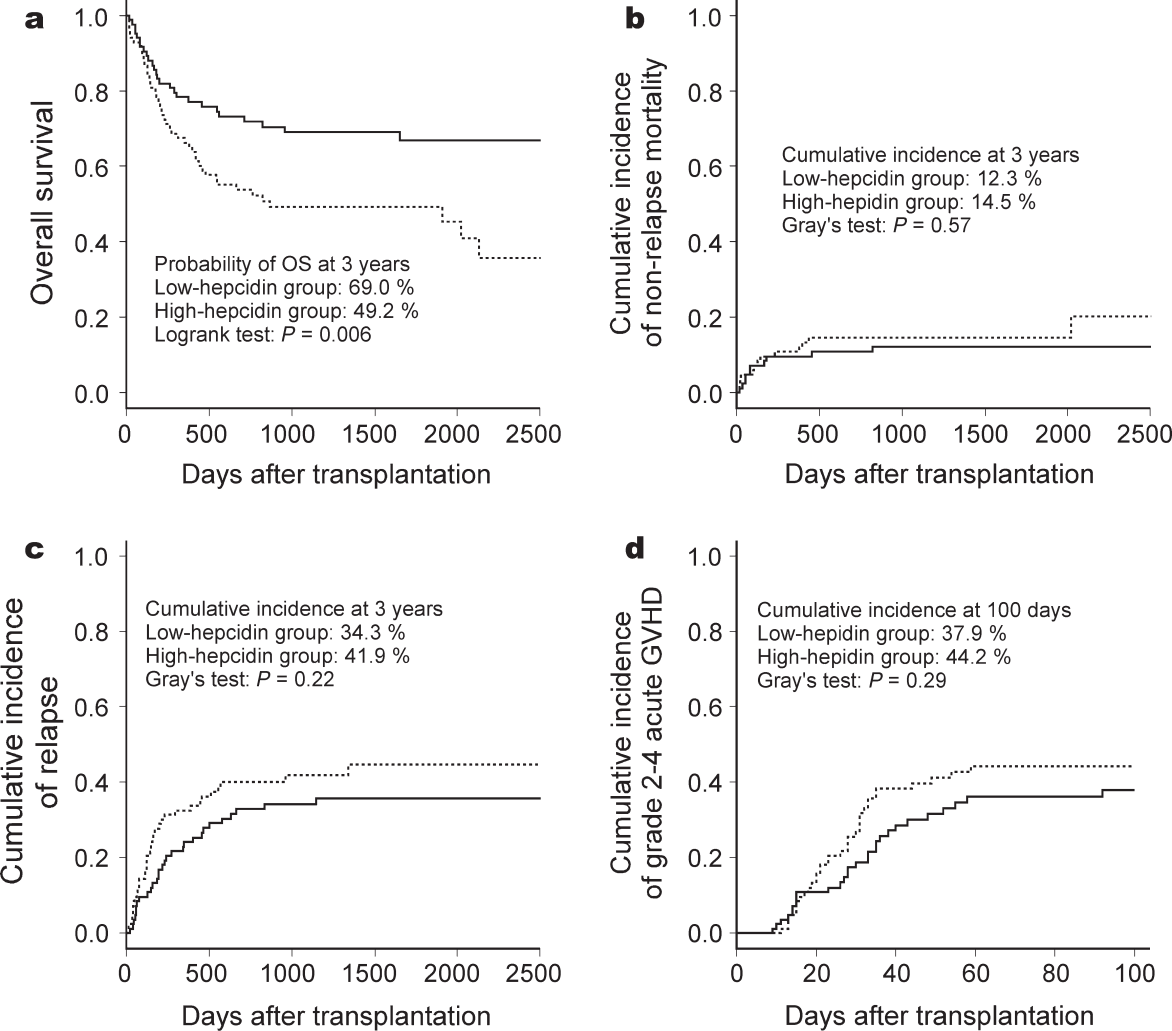
Outcome of allogeneic hematopoietic stem cell transplantation in patients with hematological malignancies stratified by pretransplant hepcidin‐25 levels. Patients were divided into two groups; solid lines indicate the low‐hepcidin group (<35 ng/mL), and broken lines indicate the high‐hepcidin group (≥35 ng/mL). (a) Overall survival (OS). (b) Cumulative incidence of nonrelapse mortality. (c) Cumulative incidence of relapse. (d) Cumulative incidence of grade 2–4 acute graft‐versus‐host disease (GVHD).

Median follow‐up time after allo‐HSCT among survivors was 46.8 months. During the follow‐up period, 16 patients relapsed and 11 died without relapse in the low‐hepcidin group, whereas 30 relapsed and 14 died without relapse in the high‐hepcidin group. The actual causes of nonrelapse mortality (NRM) are listed in Table S1. Eight and nine patients in the low‐hepcidin and high‐hepcidin groups, respectively, received second allo‐HSCT without recovery (Table S2). At 3 years, the patients in the high‐hepcidin group had a significantly lower OS than those in the low‐hepcidin group (49.2 vs. 69.0%, respectively; *P* = 0.006) (Fig. 1, panel a). No significant difference was observed between these groups in the cumulative incidences of NRM, disease relapse, or grade 2−4 acute GVHD (Fig. 1, panels b−d). On univariate analysis, pretransplant serum hepcidin‐25 ≥ 35 ng/mL, male sex, and high‐risk disease status were significant risk factors for inferior OS, whereas patient age, disease category (myeloid or lymphoid), source of stem cells, ABO matching, conditioning regimens, and GVHD prophylaxis methods did not affect OS (Table 2). Multivariate analysis revealed that pretransplant serum hepcidin‐25 level, sex and disease status were independently associated with OS (Table 2).

**Table 2:** Univariate and multivariate analyses of overall survival

Category	Univariate		Multivariate	
	HR	*P* value	HR	*P* value
Hepcidin‐25				
<35 ng/mL	1		1	
≥35 ng/mL	1.94	**0.007**	2.01	**0.005**
Age				
<50	1			
≥50	1.2	0.44		
Sex				
Male	1		1	
Female	0.54	**0.015**	0.52	**0.008**
Disease				
Myeloid	1			
Lymphoid	1.01	0.98		
Risk				
Standard	1		1	
High	2.31	**<0.001**	2.37	**<0.001**
Source of stem cells				
HLA‐matched related	1			
HLA‐mismatched related	1.66	0.3		
Unrelated BM/PB	1.54	0.23		
Unrelated CB	1.47	0.32		
ABO blood‐type matching				
Matched	1			
Mismatched	0.81	0.37		
Conditioning				
Myeloablative	1			
Reduced intensity	0.97	0.88		
GVHD prophylaxis				
Tacrolimus	1			
Cyclosporine	0.43	0.069		

The Cox proportional‐hazard model was applied to calculate hazard ratios (HRs) and *P* values. *P* values <0.05 are shown in bold‐type. HLA, human leukocyte antigen; BM, bone marrow; PB, peripheral blood; CB, cord blood; GVHD, graft‐versus‐host disease; tacrolimus‐based, tacrolimus with or without other agents; cyclosporine‐based, cyclosporine with or without other agents.

### Engraftment

**Figure 2:**
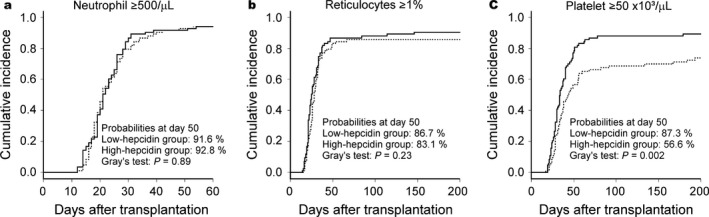
Outcome of allogeneic hematopoietic stem cell transplantation in patients with hematological malignancies stratified by pretransplant hepcidin‐25 levels. Solid lines indicate the low‐hepcidin group (<35 ng/mL), and broken lines indicate the high‐hepcidin group (≥35 ng/mL). (a) Neutrophil engraftment. (b) Reticulocyte engraftment. (c) Platelet engraftment.

Next, we analyzed the incidences of engraftment according to pretransplant hepcidin‐25 levels. No significant differences were observed in neutrophil and reticulocyte engraftments between the high‐ and low‐hepcidin groups (Fig. 2, panels a and b). In contrast, platelet engraftment was significantly lower in the high‐hepcidin group than in the low‐hepcidin group (Figure 2, panel c); the probability of platelet engraftment at day 50 in the high‐hepcidin group was 56.6% compared to 78.2% in the low‐hepcidin group (*P* = 0.002). On univariate analyses, only unrelated cord blood transplantation was a significant risk factor for inferior neutrophil and reticulocyte engraftments (Table 3). As for platelet engraftment, pretransplant serum hepcidin‐25 levels ≥35 ng/mL, high‐risk disease status, and unrelated cord blood transplantation were significant risk factors for inferior engraftment on univariate analysis; all these factors in addition to male sex of the recipient were significant risk factors for inferior engraftment on multivariate analysis (Table 3).

**Table 3:** Univariate and multivariate analysis of engraftment

Categories	Neutrophils ≥500				Reticulocytes ≥1%				Platelets ≥50,000			
	Univariate		Multivariate		Univariate		Multivariate		Univariate		Multivariate	
	HR	*P* value	HR	*P* value	HR	*P* value	HR	*P* value	HR	*P* value	HR	*P* value
Hepcidin												
<35 ng/mL	1		1		1		1		1		1	
≥35 ng/mL	1.04	0.79	1.02	0.9	0.91	0.55	0.89	0.49	0.59	**0.003**	0.62	**0.008**
Age (years)												
<50	1				1				1			
≥50	1.02	0.92			1.04	0.83			1.03	0.85		
Sex												
Male	1				1				1		1	
Female	1.01	0.95			1.06	0.71			1.36	0.077	1.44	**0.037**
Disease												
Myeloid	1				1				1			
Lymphoid	1.1	0.57			1.22	0.25			0.91	0.59		
Risk												
Standard	1				1				1		1	
High	0.8	0.17			0.94	0.7			0.65	**0.021**	0.67	**0.031**
Sources of stem cells												
HLA‐matched related	1		1		1		1		1		1	
HLA‐mismatched related	1.09	0.79	1.08	0.82	1.22	0.56	1.26	0.58	0.13		0.7	0.33
Unrelated BM/PB	0.99	0.98	0.99	0.96	1.2	0.42	1.21	0.4	0.77	0.25	0.85	0.49
Unrelated CB	0.55	**0.014**	0.55	**0.013**	0.47	**0.003**	0.48	**0.003**	0.36	**<0.001**	0.37	**<0.001**
ABO matching												
Matched	1				1				1			
Mismatched	1.14	0.43			0.85	0.35			0.99	0.96		
Conditioning												
Myeloablative	1				1				1			
Reduced	1.16	0.34			1.12	0.5			1.09	0.63		
GVHD prophylaxis												
Tacrolimus	1				1				1			
Cyclosporine	1.02	0.93			0.71	0.17			1.05	0.84		

The Cox proportional‐hazard model was applied to calculate hazard ratios (HRs) and *P* values. *P* values <0.05 are shown in bold‐type. HLA, human leukocyte antigen; BM, bone marrow; PB, peripheral blood; CB, cord blood; GVHD, graft‐versus‐host disease; tacrolimus‐based, tacrolimus with or without other agents; cyclosporine‐based, cyclosporine with or without other agents.

### Comparison of pretransplant serum ferritin and hepcidin‐25 levels as predictive markers of transplant outcome

**Figure 3:**
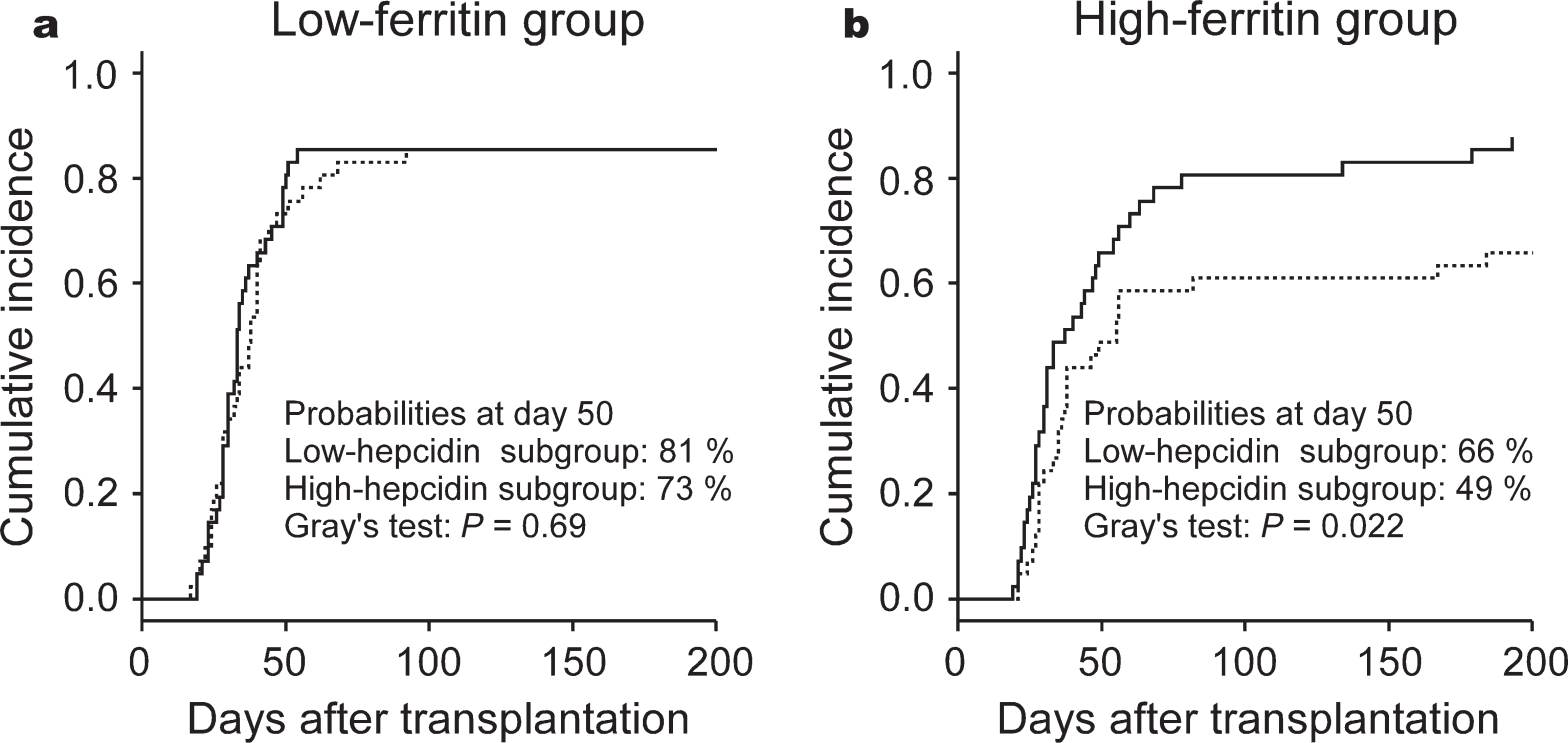
Platelet engraftment after allogeneic hematopoietic stem cell transplantation in patients with hematological malignancies stratified by pretransplant hepcidin‐25 levels. When dividing into subgroups, the cutoff values of pretransplant serum hepcidin‐25 levels were set to the median values in each group (19 ng/mL for the low‐ferritin group and 57 ng/mL for the high‐ferritin group). Solid lines indicate the low‐hepcidin subgroups, and broken lines indicate the high‐hepcidin subgroups. (a) Low‐ferritin group. (b) High‐ferritin group.

Serum hepcidin‐25 levels have been shown to positively correlate with serum ferritin level 3. Because pretransplant serum ferritin levels have been demonstrated to be an independent risk factor for the survival after allo‐HSCT 11, we compared the significance of pretransplant serum ferritin and hepcidin‐25 levels on the outcome of allo‐HSCT in our cohort. Consistent with previous reports 3, 12, pretransplant serum hepcidin‐25 levels were positively correlated with levels of serum ferritin (*R* = 0.57, *P* < 0.001, Fig. S1). We divided the patients into two groups according to the pretransplant ferritin levels; the cut‐off was set at 694 ng/mL, the median value. Consistent with previous reports 13, 14, patients in the high‐ferritin group had a significantly lower OS than those in the low‐ferritin group at 3 years post allo‐HSCT (46 vs. 72%, *P* = 0.001; Fig. S2, panel a). No significant difference was observed in the cumulative incidences of NRM, disease relapse, grade 2−4 acute GVHD (Fig. S2, panels b−d), and neutrophil and reticulocyte engraftments (Fig. S3, panels a and b) between the high‐ and low‐ferritin groups. The incidence of platelet engraftment tended to be lower in the high‐ferritin group than in the low‐ferritin group, with borderline significance (Fig. S3, panel c, *P* = 0.05 using Gray's test). When we subjected the pretransplant serum ferritin level to the multivariate analysis, serum ferritin level ≥694 ng/mL was not a significant risk factor for inferior platelet engraftment (*P* = 0.073). Furthermore, we divided the high‐ and low‐ferritin groups into high‐ and low‐hepcidin subgroups using their median values (57 ng/mL and 19 ng/mL for the high‐ and low‐ferritin groups, respectively) as cut‐offs. In the low‐ferritin group, pretransplant hepcidin‐25 levels had little effect on the incidence of platelet recovery (Fig. 3, panel a). In contrast, the incidence of platelet recovery in the high‐ferritin group was significantly lower in the high‐hepcidin subgroup than in the low‐hepcidin subgroup (Fig. 3, panel b, *P* = 0.022 using Gray's test).

## Discussion

Previous studies showed an unfavorable impact of elevated serum hepcidin‐25 levels on the survival of patients with primary myelofibrosis and non‐Hodgkin lymphoma 15, 16. In hemodialysis patients, elevated serum hepcidin‐25 levels were associated with increased risks of cardiovascular events 17. In this study, we investigated the association of pretransplant serum hepcidin levels and the outcome of allo‐HSCT. Consistent with our previous report, the median pretransplant serum hepcidin‐25 level (35 ng/mL) was higher than that of healthy volunteers 6. In this study, we found that elevated pretransplant serum hepcidin‐25 levels (≥35 ng/mL) predicted inferior OS after allo‐HSCT. We also found that elevated pretransplant serum hepcidin‐25 levels were associated with a lower incidence of platelet engraftment.

Hepcidin production by hepatocytes is controlled by various factors; for example, iron, interleukin‐6, bone morphogenetic protein 6, and oncostatin M strongly induce hepcidin production 18, 19, 20, 21, whereas hypoxia, growth differentiation factor 15, and erythroferrone suppress it 22, 23, 24. Among these factors, iron strongly induces hepcidin production. Since expression of both hepcidin and ferritin are upregulated by systemic iron overload 25, 26, it follows that serum hepcidin‐25 levels are positively correlated with those of serum ferritin 3, 12. Consistently, we observed a positive correlation between pretransplant serum levels of ferritin and hepcidin‐25 (Figure S1). In patients with hematopoietic malignancies, iron overload frequently occurs due to repeated red blood cell transfusions and/or ineffective erythropoiesis 27. In hematologic malignancies such as myelodysplastic syndromes, elevation of serum ferritin is regarded as a risk factor for mortality 28, 29. Elevation of pretransplant serum ferritin has been shown to be a prognostic factor predicting an increase in NRM and inferior OS in a number of studies including our own 13, 14. Therefore, elevated pretransplant serum hepcidin‐25 levels probably reflected iron overload and predicted inferior OS after allo‐HSCT, similar to the level of serum ferritin (Fig. 1, panel a).

In this study, we unexpectedly found that elevation of pretransplant hepcidin‐25 levels was significantly associated with lower incidence of platelet engraftment on both univariate and multivariate analyses. Since hepatic hepcidin production is suppressed by active erythropoiesis, increased pretransplant serum hepcidin‐25 levels might have reflected impaired hematopoietic environments not suitable for platelet engraftment. Additionally, the incidence of early bacterial infection was higher in the high‐hepcidin group than in the low‐hepcidin group as we previously reported 7; therefore, such infections might have negatively affected platelet engraftment in the high‐hepcidin group. In the current cohort, the cumulative incidence of early bacterial infections in the low‐ and high‐hepcidin groups were 24.5% and 44.6%, respectively; *P* = 0.027 using Gray's test. Still, the reason why only platelet engraftment, not neutrophil or erythroid engraftment, was delayed is unclear. Among the three cell lineages, the megakaryocytic may be most sensitive to an impaired hematopoietic environment or infection. Alternatively, iron overloaded status may preferentially suppress megakaryopoiesis. This hypothesis is supported by observations that iron deficiency stimulates megakaryocytic differentiation and causes thrombocytosis 30, and that reduction in iron mass by phlebotomy can increase platelet counts in patients with liver cirrhosis 31. Consistently, the incidence of platelet engraftment tended to be lower in the high‐ferritin group than in the low‐ferritin group (Fig. S3). However, elevation of pretransplant serum ferritin levels was not significantly associated with delayed platelet engraftment on multivariate analyses in our cohort. Additionally, the elevation of pretransplant serum hepcidin‐25 levels (>57 ng/mL) still had a significant adverse effect on platelet engraftment in those patients with high pretransplant ferritin levels (>694 ng/mL). Thus, the adverse impact of the elevation of pretransplant hepcidin levels on platelet recovery appeared to be stronger than that of pretransplant serum ferritin levels. It is noteworthy that the incidence of platelet recovery at day 50 was only 49% in our cohort if both pretransplant serum ferritin and hepcidin‐25 levels were high.

In summary, pretransplant serum hepcidin‐25 levels negatively impacted OS and platelet recovery after allo‐HSCT. The pretransplant serum hepcidin level can potentially be a marker for predicting delayed platelet recovery after allo‐HSCT. Since this is a relatively small, retrospective, and single institutional study, our observations require verification in future studies. The mechanism of how pretransplant serum hepcidin levels lead to a lower incidence of platelet recovery also remains to be determined.

## Conflict of Interest

The authors declare that they have no conflict of interest.

## Supporting information


**Table S1.** Cause of death.**Table S2.** Cause of second stem cell transplantation.
**Figure S1.** Correlation between pre‐transplant serum ferritin and hepcidin‐25 levels. Each circle in this double logarithmic chart represents an individual patient.
**Figure S2.** Outcome of allogeneic hematopoietic stem cell transplantation in patients with hematological malignancies stratified by pre‐transplant serum ferritin levels.
**Figure S3.** Outcome of allogeneic hematopoietic stem cell transplantation in patients with hematological malignancies stratified by pre‐transplant hepcidin‐25 levels.
