# Supplementary material for: High pretransplant hepcidin levels are associated with poor overall survival and delayed platelet engraftment after allogeneic hematopoietic stem cell transplantation
Source: Cancer Med. 2016 Dec 1;6(1):120–8. doi: 10.1002/cam4.974 (PMC5269567; doi:10.1002/cam4.974)
Supplement: Supplementary file 1 — Table S1. Cause of death.Table S2. Cause of second stem cell transplantation. Figure S1. Correlation between pre‐transplant serum ferritin and hepcidin‐25 levels. Each circle in this double logarithmic chart represents an individual patient. Figure S2. Outcome of allogeneic hematopoietic stem cell transplantation in patients with hematological malignancies stratified by pre‐transplant serum ferritin levels. Figure S3. Outcome of allogeneic hematopoietic stem cell transplantation in patients with hematological malignancies stratified by pre‐transplant hepcidin‐25 levels. [file CAM4-6-120-s001.docx]

Supplementary Table S1. Cause of death.

| Category | | Low-hepcidin Group | High-hepcidin Group |
| --- | --- | --- | --- |
| Relapse | | 16 | 30 |
| NRM |  | 11 | 14 |
|  | Infection | 4 | 4 |
|  | IPS | 1 | 2 |
|  | Organ failure | 2 | 1 |
|  | VOD | 1 | 1 |
|  | Bleeding | 0 | 2 |
|  | Chronic GVHD | 2 | 0 |
|  | Others | 1 | 4 |
| Total | | 27 | 44 |

Values indicate number of patients. NRM, non-relapse mortality; IPS, idiopathic pneumonia syndrome; VOD, venous occlusive disease; GVHD, graft-versus-host disease.

Supplementary Table S2. Cause of second stem cell transplantation.

| Category |  | Low-hepcidin group | High-hepcidin group |
| --- | --- | --- | --- |
| Graft failure |  | 1 | 5 |
| Relapse |  | 7 | 3 |
| Donor cell leukemia |  | 0 | 1 |
| Total |  | 8 | 9 |

Values indicate number of patients.


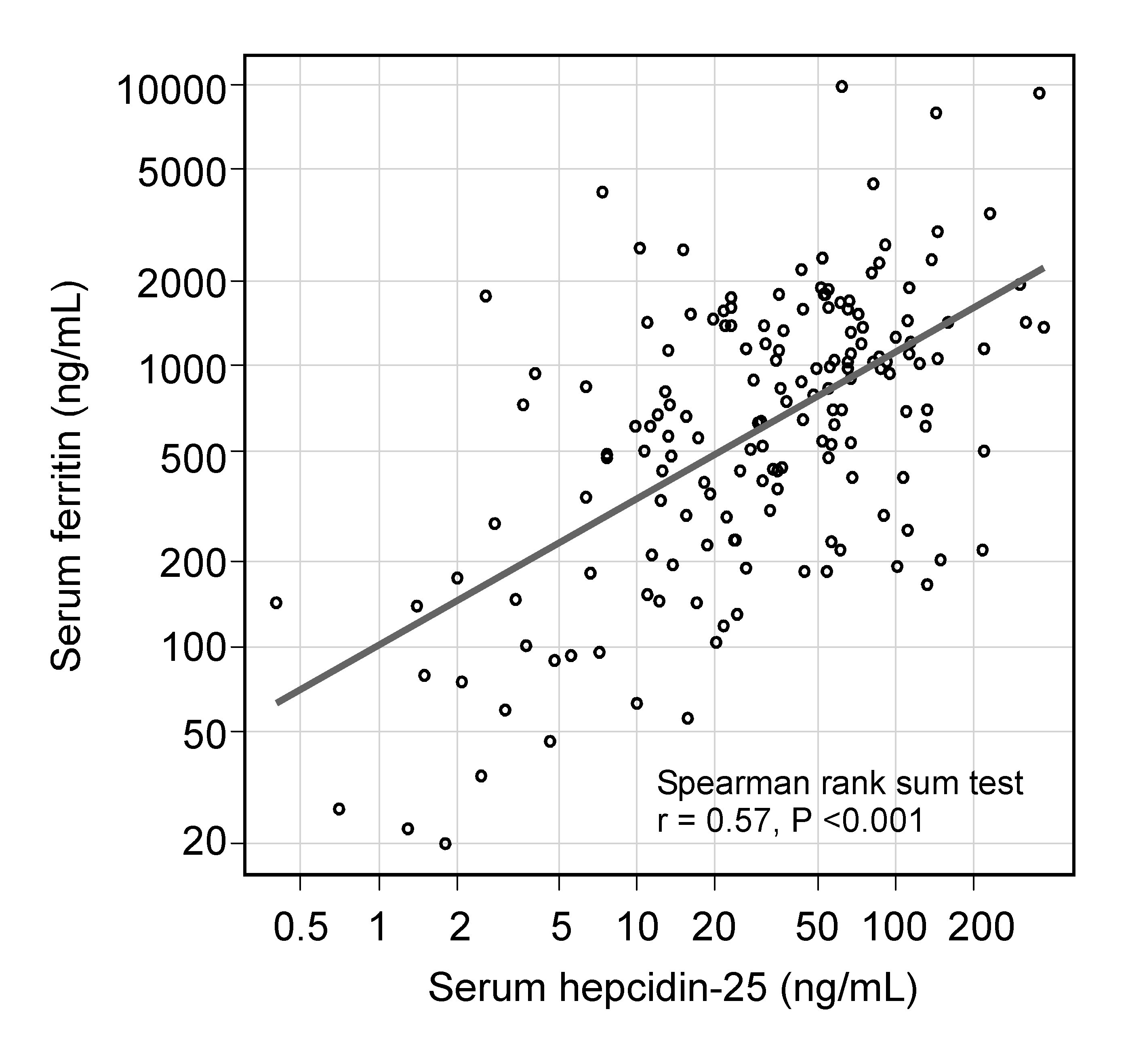


Supplementary Figure S1. Correlation between pre-transplant serum ferritin and hepcidin-25 levels. Each circle in this double logarithmic chart represents an individual patient.


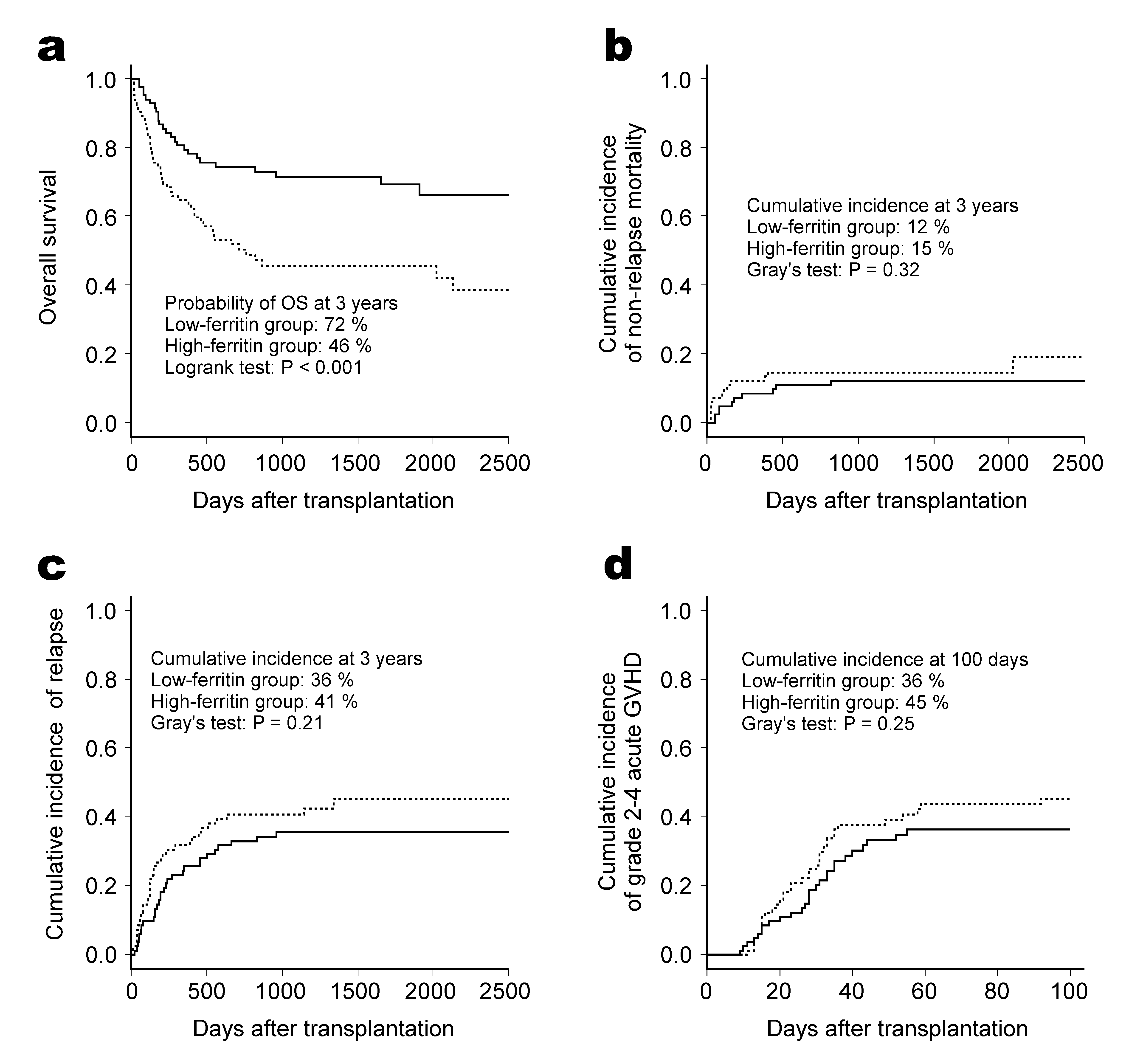


Supplementary Figure S2. Outcome of allogeneic hematopoietic stem cell transplantation in patients with hematological malignancies stratified by pre-transplant serum ferritin levels. Patients were divided into two groups; solid lines indicate the low-ferritin group (<694 ng/mL), and broken lines indicate the high-ferritin group (≥694 ng/mL). (a) Overall survival. (b) Cumulative incidence of non-relapse mortality. (c) Cumulative incidence of relapse. (d) Cumulative incidence of grade 2 to 4 acute graft-versus-host disease (GVHD).


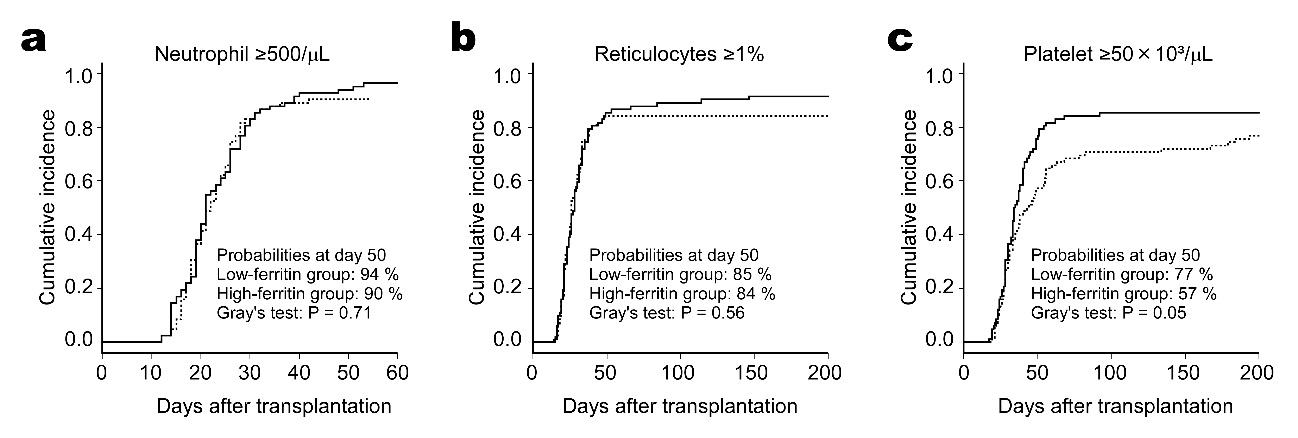


Supplementary Figure S3. Outcome of allogeneic hematopoietic stem cell transplantation in patients with hematological malignancies stratified by pre-transplant hepcidin-25 levels. Solid lines indicates the low-ferritin group (<694 ng/mL), and broken lines indicates the high-ferritin group (≥694 ng/mL). (a) Neutrophil engraftment. (b) Reticulocyte engraftment. (c) Platelet engraftment.
